# Entrapped Sediments as a Source of Phosphorus in Epilithic Cyanobacterial Proliferations in Low Nutrient Rivers

**DOI:** 10.1371/journal.pone.0141063

**Published:** 2015-10-19

**Authors:** Susanna A. Wood, Craig Depree, Logan Brown, Tara McAllister, Ian Hawes

**Affiliations:** 1 Cawthron Institute, Nelson, New Zealand; 2 Environmental Research Institute, University of Waikato, Hamilton, New Zealand; 3 NIWA, Hamilton, New Zealand; 4 Horizons Regional Council, Palmerston North, New Zealand; 5 Waterways Centre for Freshwater Management, University of Canterbury, Christchurch, New Zealand; INRA, FRANCE

## Abstract

Proliferations of the benthic mat-forming cyanobacteria *Phormidium* have been reported in rivers worldwide. *Phormidium* commonly produces natural toxins which pose a health risk to animal and humans. Recent field studies in New Zealand identified that sites with *Phormidium* proliferations consistently have low concentrations of water column dissolved reactive phosphorus (DRP). Unlike other river periphyton, *Phormidium* mats are thick and cohesive, with water and fine sediment trapped in a mucilaginous matrix. We hypothesized that daytime photosynthetic activity would elevate pH inside the mats, and/or night time respiration would reduce dissolved oxygen. Either condition could be sufficient to facilitate desorption of phosphates from sediment incorporated within mats, thus allowing *Phormidium* to utilize it for growth. Using microelectrodes, optodes and pulse amplitude modulation fluorometry we demonstrated that photosynthetic activity results in elevated pH (>9) during daytime, and that night-time respiration causes oxygen depletion (<4 mg L^-1^) within mats. Water trapped within the mucilaginous *Phormidium* mat matrix had on average 320-fold higher DRP concentrations than bulk river water and this, together with elevated concentrations of elements, including iron, suggest phosphorus release from entrapped sediment. Sequential extraction of phosphorus from trapped sediment was used to investigate the role of sediment at sites on the Mangatainoka River (New Zealand) with and without *Phormidium* proliferations. Deposition of fine sediment (<63 μm) was significantly higher at the site with the most extensive proliferations and concentrations of biological available phosphorus were two- to four- fold higher. Collectively these results provide evidence that fine sediment can provide a source of phosphorus to support *Phormidium* growth and proliferation.

## Introduction

Proliferations of freshwater benthic mat-forming cyanobacteria are being reported with increasing frequency worldwide [1–3]. The species responsible for these proliferations produce a range of natural toxins (cyanotoxins) which pose a health risk to humans and animals. Multiple animal fatalities have been associated with ingestion of benthic cyanobacteria mats [4–7]. Studies also indicate significant ecological impacts including toxicity to aquatic organisms [8,9], and shifts in macroinvertebrate communities [10].

*Phormidium* is one of the most commonly reported problematic toxic benthic cyanobacteria in rivers [5,11]. Under favourable conditions, *Phormidium* can form expansive black / brown leathery mats that may cover the entire substrate and stretch for many tens of kilometres [12]. *Phormidium* has been studied in New Zealand rivers for the past decade and data suggests that proliferations are usually observed during stable flow conditions, and sites with the greatest coverage generally have low water column concentrations of DRP (i.e.< 0.01 mg L^-1^) [13,14]. Although *Phormidium* proliferation occur in rivers in other countries [3,5] little is known about the nutrient status of these sites. Phosphorus is essential for cyanobacterial growth, and proliferations, or blooms, are commonly associated with elevated concentrations of this nutrient [15]. To explain this apparent discrepancy, the aim of this study was to explore the possibility that *Phormidium* mats may have alternate sources of phosphorus.

The thick cohesive mat growth forms seen in dense *Phormidium* proliferations can be many millimetres thick and they may therefore be functionally different to many other types of river periphyton communities, such as trailing green algal filaments and spongy stalked diatom mats. *Phormidium* mats form a coherent, mucilaginous matrix that adheres to the underlying substrate as a cohesive layer and interaction between the river water and the water phase inside the mat is restricted. In general, exchange of materials between gelatinous biofilms and overlying water is restricted by diffusion within the mat and through the diffusive boundary layer that separates the mat from the bulk water phase [16]. In other thick, mucilaginous cyanobacterial mats, this isolation allows geochemical conditions within mats to diverge from those in the overlying water, particularly with respect to biologically active materials [17,18]. Steep gradients of variables such as dissolved oxygen concentration and pH, that are dynamic on day-night cycles, are common [19]. While most observations of microbial mat biogeochemical profiles have been made on soft sediments in slow-flowing or static waters, similar considerations apply to thick river biofilms that overlie impermeable rock substrates.

A feature of most *Phormidium* mats is a thin layer of fine sediment at the substrate / mat interface [20]. *Phormidium* filaments secrete extracellular polymeric substances (EPS) as they grow [21] and fine-grained sediment particles that are continually washed across the mat surface stick to the EPS and are incorporated into the mat matrix. *Phormidium* filaments are very motile [22], and can likely use their motility to stay above the trapped and bound particles and thus gradually migrate fine sediments into the lower mat matrix. Sedimentation in rivers is challenging to measure, but sediment deposition estimates can be quantified with sediment traps placed in the substratum [23]. Wood and Bridge [24] used sediment traps and showed a positive relationship between quantity of deposited fine sediment and propensity of sites to support *Phormidium* proliferations in the Maitai River (Nelson, New Zealand). Phosphorus is present in sediments in a number of chemical forms that range in bioavailability from loosely sorbed phosphate that may be considered readily bioavailable, to phosphorus bound to metal oxide phases, through to phosphorus that is tightly bound in the lattice of insoluble apatite minerals and therefore not bioavailable [25,26].

We hypothesize that the modification of environmental conditions within *Phormidium* mats can mobilize phosphates bound to entrapped sediments. There are two possible scenarios that may facilitate desorption of phosphates within the mats: (1) during daytime, a photosynthesis-driven increase in pH may occur within the mat, as use of bicarbonate results in a local accumulation of hydroxide [27,28,29]; or (2) depletion of oxygen at night through aerobic respiration [30,31]. Under these conditions iron, aluminium and manganese minerals can be solubilised, leading to the release of phosphates bound to those minerals. This ‘alternate’ source of phosphorus may at least partially explain how *Phormidium* proliferations are able to attain a high biomass in relatively low nutrient rivers.

In this study we aimed to test the hypotheses that: (i) daytime photosynthetic activity by *Phormidium* would elevate pH inside the mats, and/or night time respiration would reduce dissolved oxygen sufficiently to facilitate desorption of loosely bound phosphates from sediment incorporated within mats; and (ii) *Phormidium* proliferations would be more likely to occur at sites with higher depositional mass loads of bioavailable phosphorus associated with fine sediment. To test these hypotheses a ‘river mesocosm channel’ containing rocks with thick *Phormidium* mats was set up and microelectrodes and optodes used to measure pH and dissolved oxygen (DO). River water and ‘within *Phormidium* mat’ water samples were collected and analysed for DRP and elemental composition. Sediment traps and sequential extraction of phosphorus were used, respectively, to investigate sedimentation rates and biologically available phosphorus at three sites on the Mangatainoka River (New Zealand) experiencing varying intensities of *Phormidium* proliferations.

## Methods

### Study site

**Fig 1:**
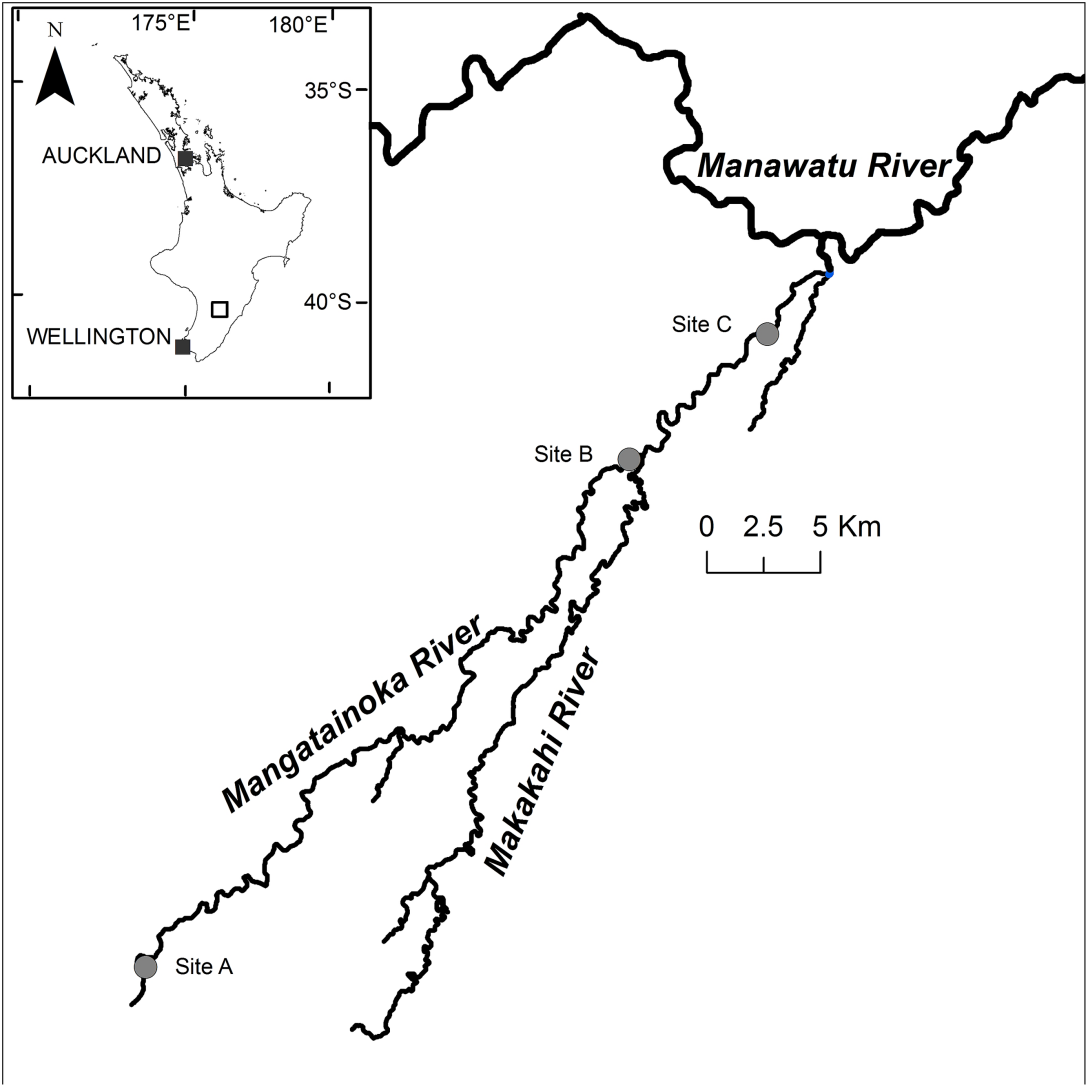
Location of study site (B) and sediment traps sites (A, B and C) in the Mangatainoka River, New Zealand.

The upper catchment of the Mangatainoka River (New Zealand) drains a largely native forest covered region of the northern Tararua Ranges (Fig 1). As it flows northwards, the surrounding land use changes to mostly sheep and beef (ca. 50%), and dairy (ca. 30%). *Phormidium* proliferations have been recorded in the lower reaches of the river since monitoring began in 2011 [14]. The field experiment commenced 11 March 2014 in the lower Mangatainoka River (site B, 40°28'36" S, 175°47'14" N; Fig 1). The experiment was set up in a 200 m long riffle (avg. width 14.5 m, depth 0.15 m). Well developed *Phormidium* mats covered 50% of the substrate.

Permission to work at all sites in the Mangatainoka River was given by the Horizon Regional Council. The field studies did not involve endangered or protected species.

### Sample collection and analysis

Water samples were collected mid-river (200 mL) and from within the *Phormidium* mats (30 mL) every 2 to 4 h. To obtain water isolated from within the *Phormidium* mats—hereafter referred to as ‘within mat water’, approximately 10 rocks with thick *Phormidium* mats were lifted gently from the river and surface water drained for ca. 30 s. Mats were scraped into a beaker (500 mL), held lightly with a spatula, and residual surface water drained. The mats were then gently pressed repeatedly against the beaker wall using a stainless steel spatula to obtain within mat water.

Water samples were filtered (GF/C filter, Whatman) and aliquots (10 to 50 mL) taken for DRP analysis. These were stored on ice in the field and frozen (-20°C) within 10 h of sampling. Analysis of DRP was undertaken using a Lachat Quick Chem^®^ Flow Injection Analyser (FIA+ 8000 Series; Zellweger Analytics, Inc., Milwaukee, WI, USA) according to methods given in APHA, AWWA, and WEF [32] (limit of detection 0.001 mg L^-1^). A second filtered subsample (10 mL) was preserved (2% nitric acid), stored chilled (4°C), and elemental concentrations measured using an inductively coupled plasma mass spectrophotometer (ICP-MS; Perkin Elmer ELAN DRC II; Perkin Elmer Analytical Instruments, USA) at the University of Waikato Mass Spectrometry Facility (Hamilton, New Zealand). Paired student t-tests (Microsoft Excel) were used to determine statistically significant differences among river and within mat water DRP and element concentrations.

### Continuous loggers and experimental set up

Temperature, pH and DO were recorded at a mid-river site at 5 min intervals between 09:30h, 11 March 2014 and 09:30h, 13 March 2014 (EXO2 water quality sonde, YSI, USA). Photosynthetically active radiation (PAR) measurements were recorded just below the water surface over the same period (5-min intervals) using a Li-CorLi192 PAR sensor coupled to a Li-Cor Li1400 meter in a waterproof housing.

A ‘river mesocosm chamber’ as described in Hickey [33], was set up at the edge of the river and used for fine-scale measurements within *Phormidium* mats. The chamber was made of clear poly(methyl)methacrylate (‘Perspex’), with external dimension of 0.8 m × 0.24 m and a volume of 8.5 L. The chamber was modified from that described in Hickey [33] by removal of the lid and a submersible 12V pump (Rule, USA) with a 2.2 L s^-1^ pumping capacity was used to maintain an open circuit flow velocity of ca. 0.2 m s^-1^ through the chamber. Two boulders (ca. 150 mm diameter) with thick (3–4 mm) *Phormidium* mats were placed in the chamber so that they were fully submerged.

**Fig 2:**
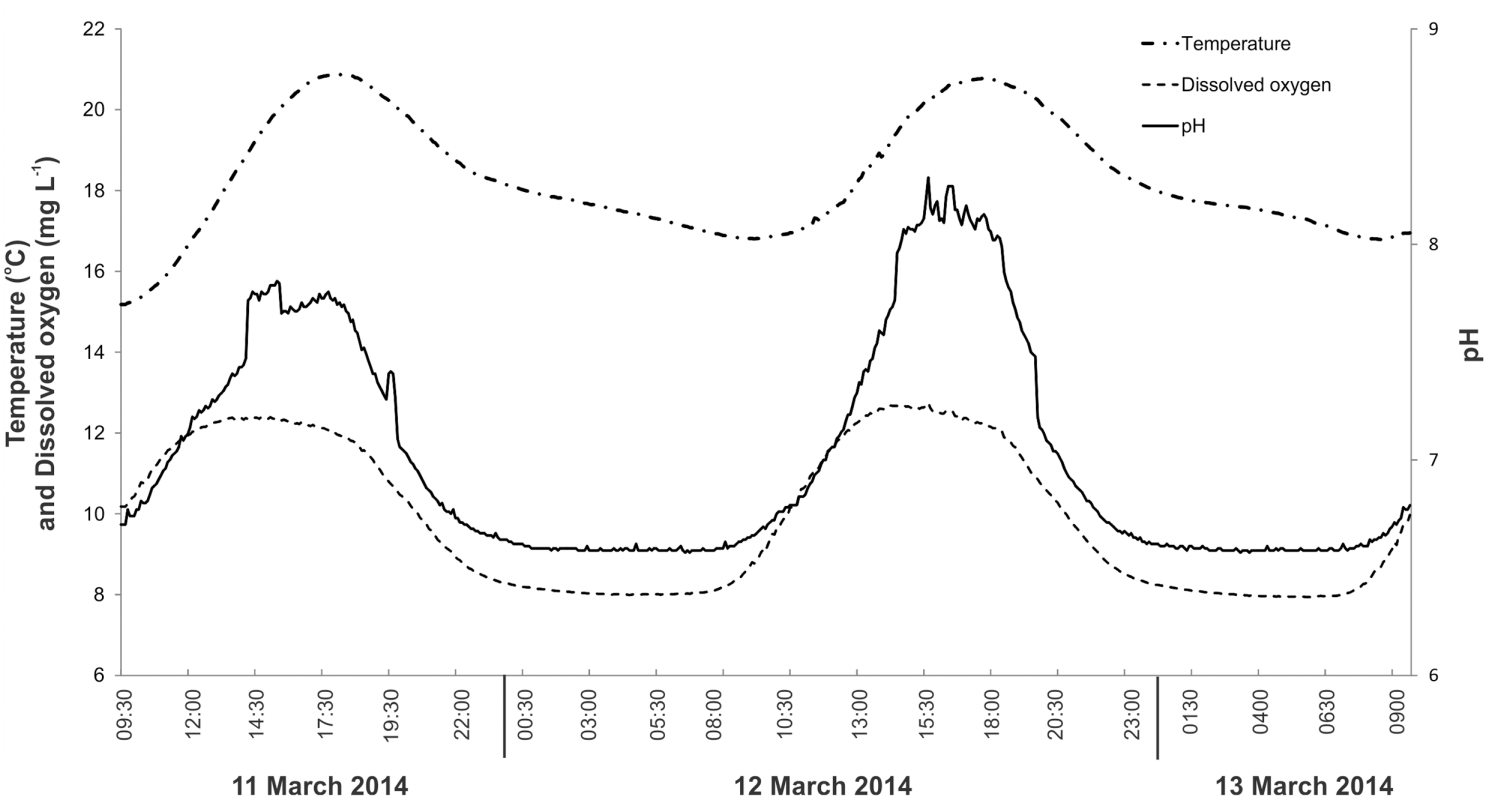
Dissolved oxygen (DO) concentrations (1-min intervals) measured within *Phormidium* mats in the ‘mesocosm chamber’ in the Mangatainoka River (site B, 11 to 13 March 2014). Solid arrows indicated where the DO optode was repositioned. Open arrows indicates where the pump which maintained the flow across the chamber failed.

A PreSens oxygen optode (50 μm dia.), recording onto a PreSens AS1 data logger via a TX3 signal processor (PreSen, Denmark) was used to obtain *in situ* oxygen measurements at 1 min intervals between 12:30h on 11 March 2014 and 09:40h on 13 March 2014. The optode was lowered into the *Phormidium* mat in 100 μm steps using a micromanipulator until positioned approximately 1 mm beneath the mat surface. Calibration of the optode was using fully saturated river water and water deoxygenated using sodium dithionite. Some movement of mat occurred during the experiment, and the optode was repositioned four times (Fig 2) to ensure that it remained embedded in a cohesive part of the mat.

Depth profiles of pH were measured through *Phormidium* mats using a Unisense pH micro-electrode (pH-50, 50 μm dia.) connected to a Unisense Microsensor Meter, at 14:30h, 14:45h, 15:45h, 16:00h and 17:30h (11 March 2014). The micro-electrode was lowered through the *Phormidium* mat and measurement taken in 100 μm intervals using a micromanipulator. *In situ* pH measurements were also taken between 07:40h and 15:00h (12 March 2014) at ca. 1 h intervals using a Unisense pH semi-microelectrode housed within a 1.1 mm external diameter syringe needle (pH-N) connected to a Unisense Underwater Meter. The micro-electrode was positioned inside the mat using a micromanipulator. Linear calibrations of the mV readings were performed in standard buffers at pH 7.0, 10.0 and 11.0, at *in situ* temperatures.

The diurnal pattern of photosynthesis was followed using Pulse Amplitude Modulated fluorescence (PAM). A monitoring-PAM sensor (Walz Mess- und Regeltechnik, Germany), equipped with white excitation LEDs, was placed mid-river and directed at a cobble with a cover of *Phormidium*. The instrument was configured to measure at 5-min intervals between 07:40h, 12 March 2014 and 09:25h, 13 March 2014. PAM sensors estimate photosynthetic activity by comparing fluorescence yield of photosystem II (PSII) under ambient irradiance (*F*) and after application of a saturating pulse (*F*_m_´). The yield of PSII (*Y*_II_), a proxy for the quantum yield of photosynthesis, is calculated as (*F*_m_´- *F*)/(*F*_m_´) (see Schreiber 2004 for review of the saturation pulse method) and the product of this yield and the ambient irradiance is the relative electron transport rate (rETR), an estimate of relative photosynthetic activity [34].

### Sediment trap deployment and analysis

Sediment traps (nine per site) were deployed at the upstream end of a riffle at three locations on the Mangatainoka River on 22 March 2014 (sites A, B and C, Fig 1). Traps were 2.2 L buckets (Sistema Plastics, New Zealand) with an internal diameter of 150 mm and depth of 135 mm. Each trap was filled with washed cobbles and gravels and buried in the river flush with the surface of the river bed. Lids were removed upon deployment and replaced prior to collection to eliminate loss of fine material [35]. Triplicate traps were collected from each site on 31 March, 7 April and 14 April 2014.

Particle size distribution of the trapped sediment was determined using a wet sieving tower (Glenammer Engineering, UK) of decreasing mesh sizes (1 mm, 500 μm, 250 μm, 125 μm and 63 μm). Particles greater than 1 mm were not analysed, as they were not considered relevant to *Phormidium* as we have never observed such large particles inside mats. The wash water, containing the < 63 μm fraction was collected in 20 L buckets and allowed to settle for three to five days, after which the water was siphoned off. Each size fraction was then oven dried (105°C) and weighed. The sedimentation rate as grams per square metre per day (g m^-2^ d^-1^) was calculated.

Statistical analysis of these taken data was undertaken in R [36]. Multivariate analyses showed no difference in sedimentation rates between weeks across all particle size classes (PERMANOVA [37], p>0.05). Data were therefore pooled across weeks to give an integrated measure of sedimentation for each site over the three week time period. Differences in sedimentation rates between sites for each particle size class were tested using generalised linear models (GLM) with gamma distributed errors. Data were log transformed as it was strictly positive and right skewed. Significant differences between sites were investigated using posterior pair-wise comparisons with the Tukey Honest Significant Difference (HSD) method.

### Bioavailability of phosphorus in sediment

The phosphorus fractionation method followed Lukkariet al., [38] with minor modifications. Equal portions of dried sediment from the 500 μm, 250 μm, 125 μm, 63 μm and < 63 μm fractions from the nine replicates per site were combined (i.e. giving a single pooled sample for each size fraction per site) and homogenised. A subsample of each (ca. 500 mg) was weighed and phosphorus was extracted in 40 ml of sodium chloride (NaCl; 0.46 M). Samples were mixed briefly on a vortex mixer, extracted for 1 hour (30°C, with shaking at 200 rpm) and centrifuged (3,200 × *g*, 30 min). The supernatant liquid was filtered (0.45 μm) and stored frozen (-20°C) until analysis of phosphorus as DRP. This fraction was referred to as the loosely bound phosphorus fraction. The sediment residue was then used for the next phosphorus fraction extraction, in 40 ml of 0.11 M sodium dithionite (Na_2_S_2_O_4_) in 0.11 M sodium bicarbonate (NaHCO_3_) buffer (pH 7.0). The samples were mixed briefly, extracted for 1 h (40°C, with shaking at 200 rpm), centrifuged (3,200 × *g*, 30 min), filtered (0.45 μm) and bubbled carefully with compressed air (purity 99%) to remove dithionite (which decomposes to thiosulfate and hydrogen sulfite ions). Samples were filtered (0.45 μm) and stored frozen (-20°C) until analysis as DRP. Phosphorus released by this extraction step is referred to as reductant soluble phosphorus. The final extraction of phosphorus from the sediment residue was with 40 mL of 0.1 M sodium hydroxide (NaOH) for 18 h at 40°C (at 200 rpm). The supernatant was filtered (0.45 μm) and the pH of the resulting solution adjusted to 7.0 using hydrochloric acid (HCl). The neutralised solutions were filtered (0.45 μm) and stored frozen (-20°C) until analysis as DRP. Phosphorus released by this extraction is referred to as the metallic oxide-bound phosphorus fraction. For all extractions, DRP was analysed as described above.

The aim of this analysis was to investigate whether phosphates bound to sediments entrapped within mats might be available to *Phormidium* if geochemical conditions were favourable for desorption. We therefore did not extract the apatite and calcium carbonate bound phosphorus, or refractory mineral phosphorus as these fractions are assumed to be an inert and not generally considered as bioavailable for algal growth [39]. We also acknowledge that organic phosphorus (nonreactive phosphorus, NRP), can be a major component of phosphorus in sediments [40]. Consideration of the extent to which NRP in entrapped sediment is available and contributes to *Phormidium* growth is required for a full understanding of phosphorus dynamics. However, as our focus was on the availability of sediment-bound phosphorus and this was not assessed in this study.

### Analysis of sediment isolated from within *Phormidium* mats

Separating the sediment from the *Phormidium* mats proved challenging due to the EPS matrix. Sections of mat were homogenised (2 mins) using an Ultra-Turrax probe (IKA Laboreknik, Germany), and acid digested (10% HCl, 60 min, 30°C, in a sonication bath). A silica colloid density solution (Percoll, 30%) was added to small sub-samples (ca. 0.1 mg) and centrifuged (1800 × *g*, 10 min). This achieved partial separation, but the samples were neither clean nor large enough for particle size analysis, and instead were examined under a microscope (BX51, Olympus) at 1,000× magnification to visually estimate the average particle size.

## Results

### 3.1 Temperature, pH and dissolved oxygen in bulk river water and within *Phormidium* mats

**Fig 3:**
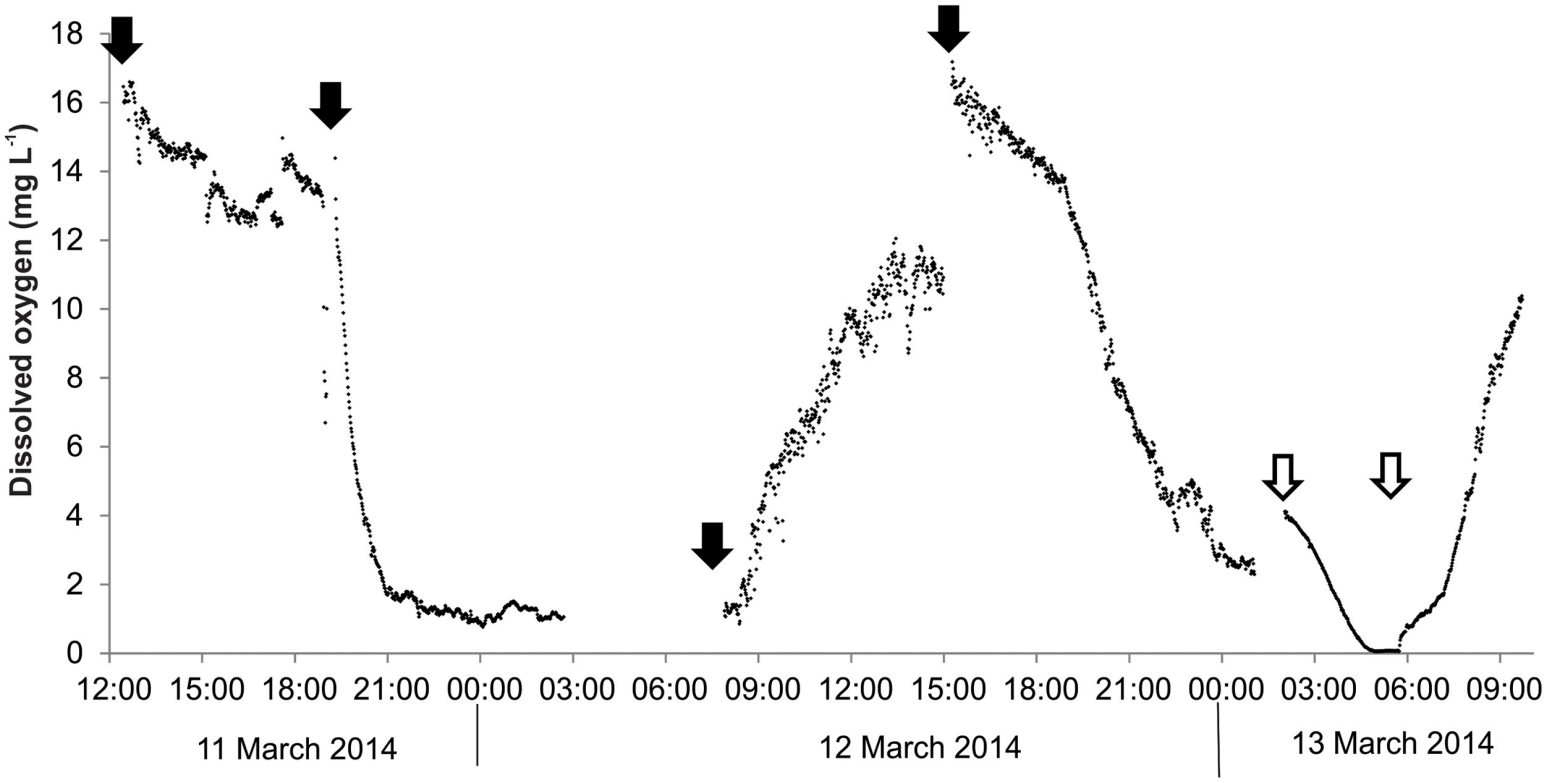
Temperature, dissolved oxygen concentration and pH mid-channel in the Mangatainoka River (site B, 11 March to 13 March 2014) measured using a water quality sonde (EXO2, YSI, USA) logging at 5-min intervals.

Over the 3-day period, the concentration of DO in the river water varied from 7.9 to 12.8 mg L^-1^, and temperature and pH ranged from 15.2°C to 20.9°C, and 6.6 to 8.3, respectively (Fig 2). All showed distinct diel variation; the maximum DO concentration occurred at approximately 14:00h, whereas maximal pH occurred between 15:00 and 17:00h. Temperature peaked in the early evening at around 18:00h (Fig 3).

Concentrations of DO within the *Phormidium* mat were elevated above bulk river water at the commencement of the experiment, and remained raised until 19:30h when they decreased to <4 mg L^-1^ at 21:00h (12 March; Fig 2). The following day DO concentrations increased from daylight onwards, and by 11:00h, bubbles of oxygen were observed at the *Phormidium* mat surface in the river mesocosm chamber. This corresponded with DO concentrations within the mat of greater than 8 mg L^-1^ (>100%; Fig 3). Dissolved oxygen concentrations declined to less than 4 mg L^-1^ by 23:00h on 13 March, prior to a pump malfunction that occurred between 02:00h to 05:30h (Fig 2). This stopped all river water flow through the chamber, resulting in DO concentrations decreasing to 0 mg L^-1^ over this period (Fig 2).

**Fig 4:**
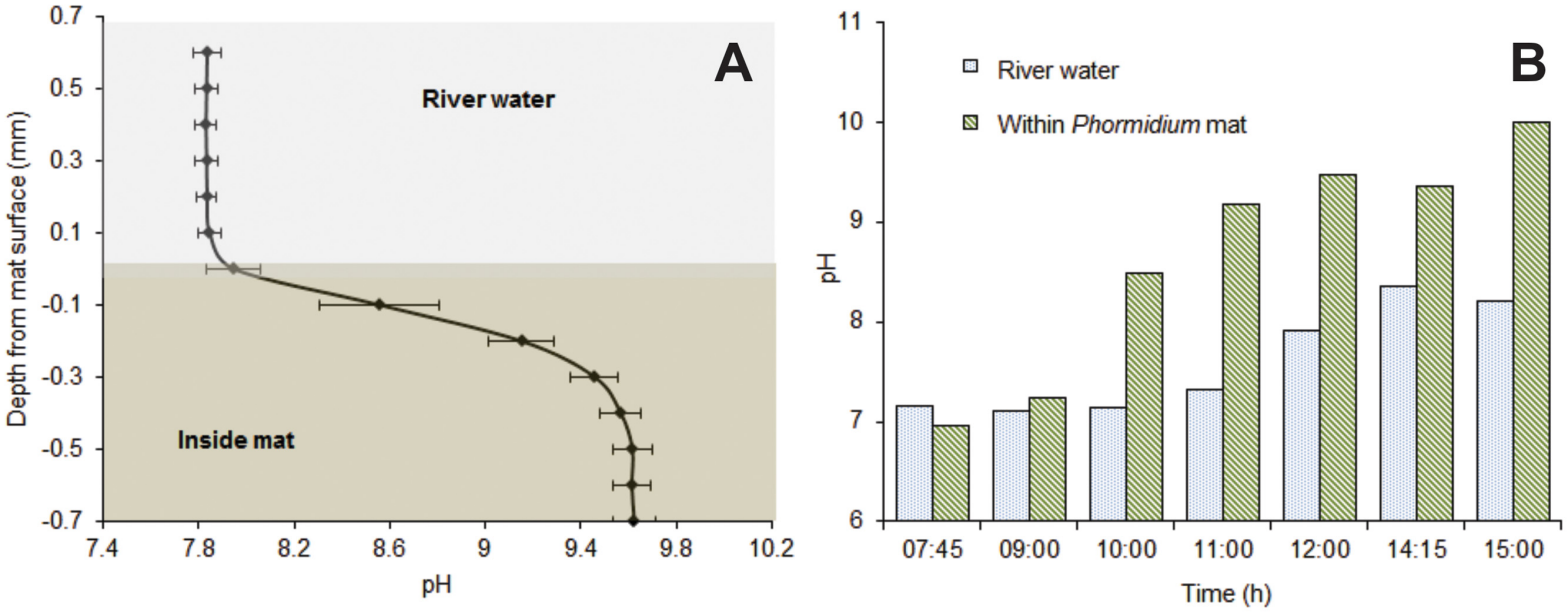
pH microelectrode data. (A) Average pH profile (n = 5) through the surface boundary layer of a *Phormidium* mat, afternoon of 11 March 2014, (B) changes in pH in river water (ca. 3 mm above the mat) and within the *Phormidium* mat during the day, 12 March 2014.

Microelectrode profiling identified that the average pH value for the bulk river water, 1 mm above the surface of the *Phormidium* mats was 7.8 (n = 5; Fig 4a). This was similar to the value measured mid-channel using the water quality sonde (Fig 3). All pH profiles through the *Phormidium* mats showed a sudden and marked increase in pH (> 9.4) at a depth of 0.1 mm to 0.2 mm. Following this there was a gradual increase in pH to a depth of approximately 0.6 mm, and thereafter it remained relatively constant at 9.6 (Fig 4a).

A semi-micro electrode was used to track pH just above (ca. 3 mm) and in the *Phormidium* mat over an 8-h period in the river mesocosm channel. At 07:30h the pH in the river water was 7.1. This remained relatively constant until 11:00h when it gradually increased to a peak of 8.4 at 14:30h (Fig 4b). The pH within the *Phormidium* mat was ca. 7.0 at 07:30h, and increased relatively quickly reaching a pH of 10.0 by the final reading at 15:00h (Fig 4b).

**Fig 5:**
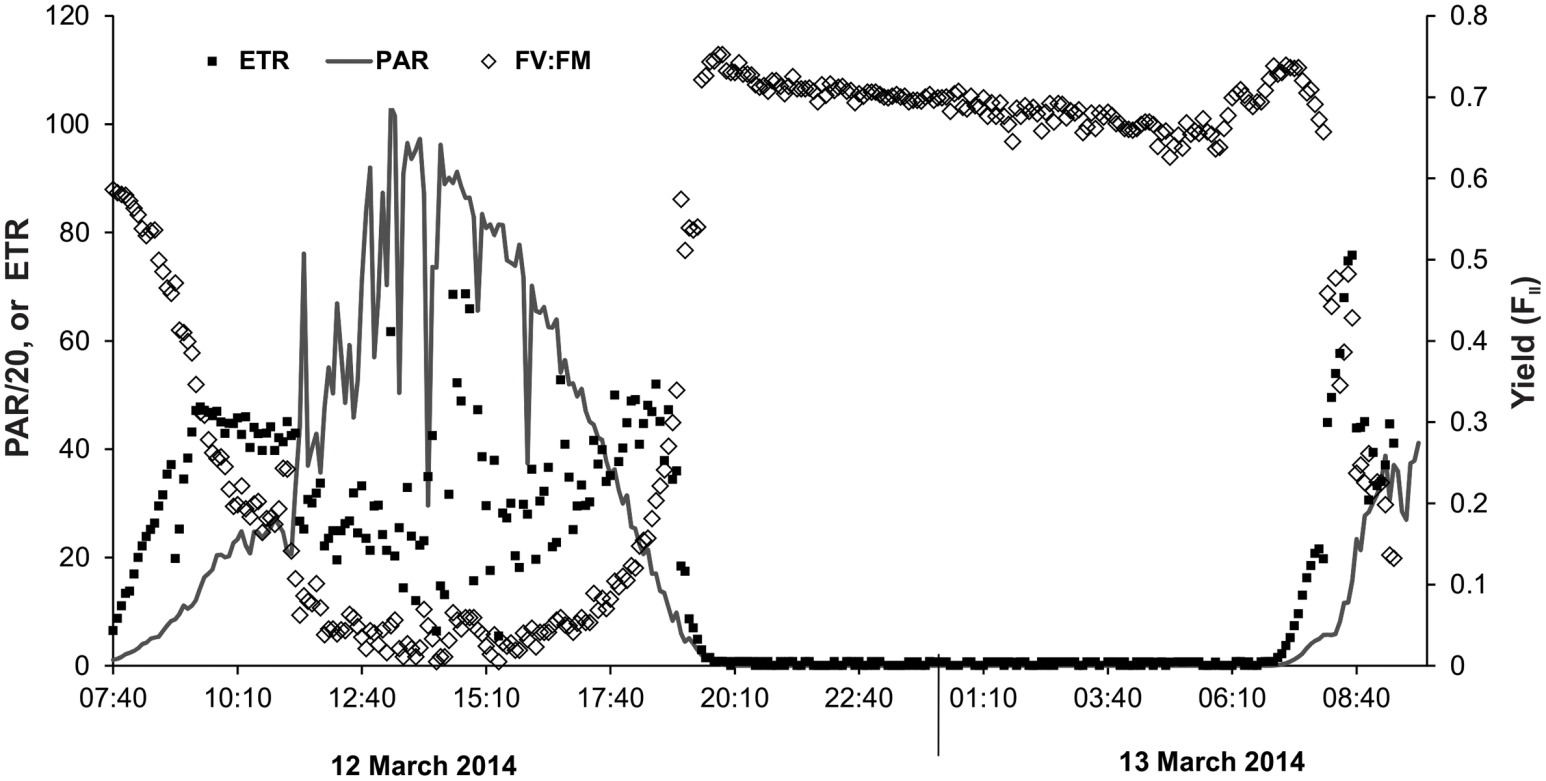
Photosynthetically active radiation (PAR; μmol photons/m^-2^ s^-1^; note values are divided by 20 to fit axis) measured just below the water surface in the Mangatainoka River (site B), and electron transfer rate (ETR; μmol electrons m^-2^ s^-1^ and yield (F_II_ [calculated as (*F*_m_´- *F*)/(*F*_m_´)]) measured on a *Phormidium* mat mid-channel in the Mangatainoka River (11 March to 13 March 2014; 5-min intervals).

Photosynthetically active radiation reached a maximum of ca. 2,000 μmol photons m^-2^ s^-1^ between 12:00h and 13:00h (Fig 5). Values varied due to sporadic cloud cover. The electron transport rate (ETR) increased steadily as PAR rose, tending towards a maximum around 09:00h (Fig 5) before decreasing until ca. 14:00h. Although quite variable, there was a general increase after 14:00h, which then decreased to zero with the onset of darkness (19:00h; Fig 5). The yield (*Y*_II_) showed the reverse trend, decreasing to less than 0.1 between 11:00 and 18:00h, and then returning to over 0.7 during darkness (Fig 5).

### Nutrient and iron concentrations

The average DRP concentration in the bulk river water was 1 μg L^-1^. Concentrations were 320-fold higher (381 μg L^-1^) in the within mat water (S1 Table). Iron concentrations were 21 times higher in the within mat compared to the river water samples (avg. 37 vs. 2.7 μg L^-1^). Within mat water concentrations were significantly higher in a further 19 of the 21 elements measured (S1 Table). The DRP concentrations remained relatively constant in the bulk river water over the sampling period, whereas there was marked variability in the concentrations of DRP in the within mat water (S2 and S3 Tables).

### Sedimentation rates and particle size distribution

**Fig 6:**
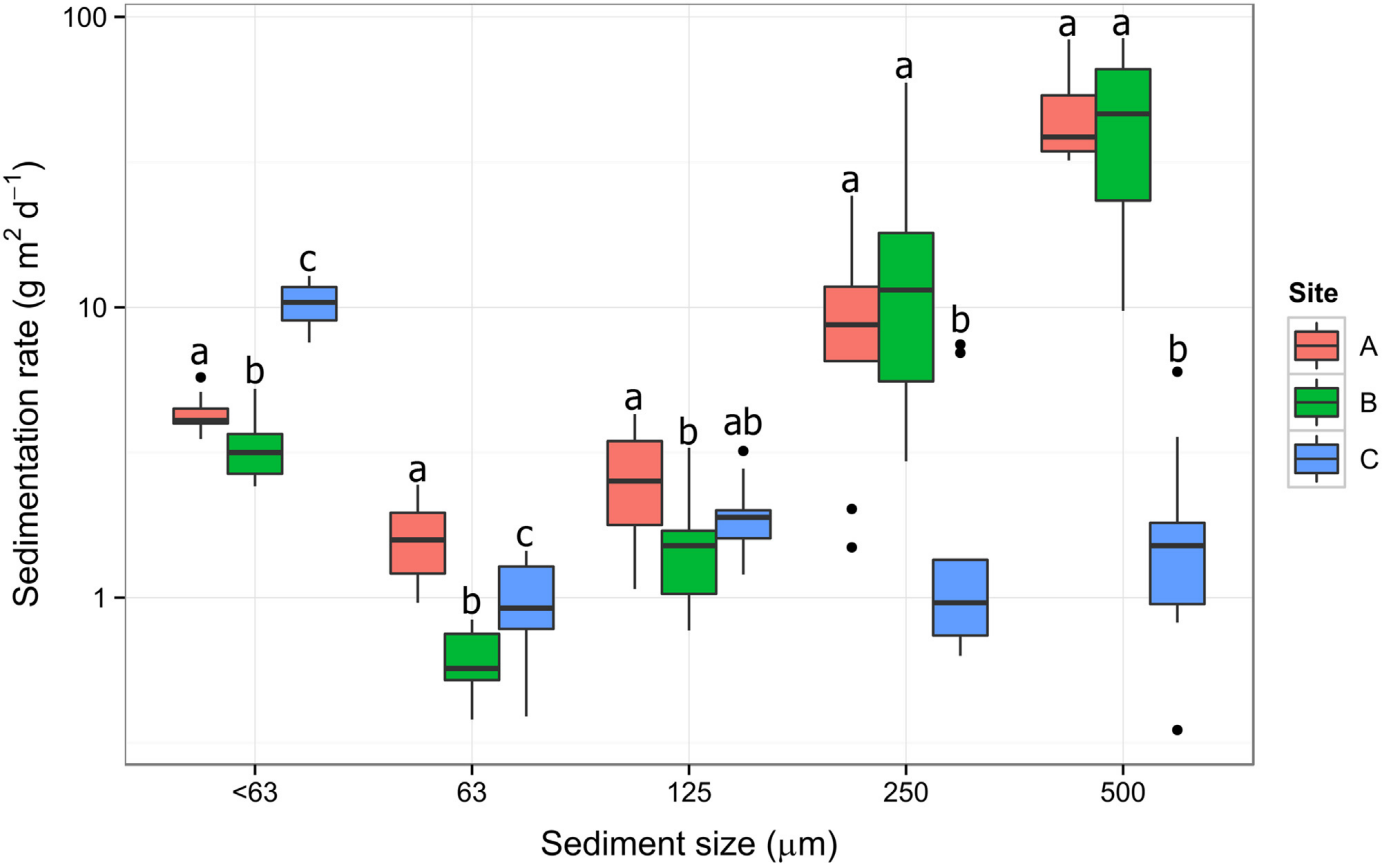
Box-plot of the sedimentation rates by size class over a 3-week period (22 March to 14 April 2014) at three sites along the Mangatainoka River. The y-axis scale is in a log10 scale to aid visualisation. Solid black line shows median, box shows 1st and 3rd quartiles, whiskers extend to the last data point within 1.5 times the inter-quartile range (n = 9). The letters above the boxes represent the results of Tukey pair wise comparisons.

The highest sedimentation rates were measured at site B (average over 3-week period, 63 g m^2^ d^-1^), followed by the upstream site A (54 g m^2^ d^-1^). Sedimentation rates were significantly different between sites for all size classes (GLM, Site p<0.05). The largest differences in sediment rates were observed for site C, having significantly more of the < 63 μm size fraction and less of the 250 and 500 μm size fraction compared to sites A and B (Fig 6).

### Phosphorus fractionation

**Fig 7:**
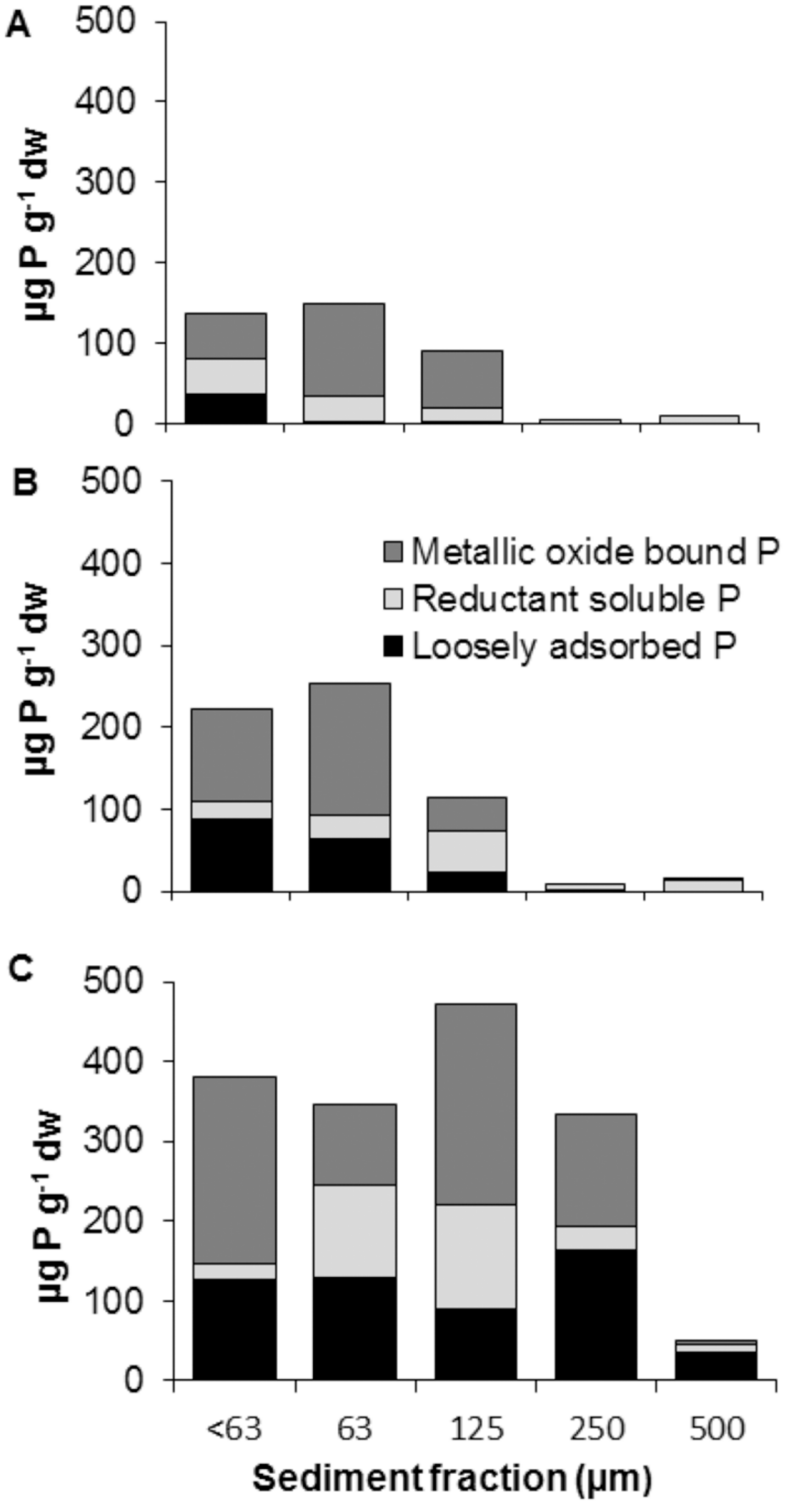
Phosphorus (P) fractionation for each sediment size fractions in a pooled sample from all sediment traps deployed at each site (n = 1). All concentrations are expressed on a dry-weight (dw) basis.

The concentrations of all analysed forms were generally greatest at site C (Fig 7), while at all three sites, the highest combined concentrations of phosphorus tended to be extracted from the < 63 μm, 63 μm and 125 μm fractions (Fig 7). Within the three smallest size classes, the most abundant phosphorus fraction was metallic oxide-bound, followed by loosely adsorbed. An exception to this was the 63 μm-fraction from the site C, where loosely adsorbed phosphorus was the most abundant fraction, followed by reductant soluble phosphorus (Fig 7). Microscopy analysis revealed that the majority of sediment particles isolated from within *Phormidium* mats were small (1–5 μm) and commonly formed agglomerations.

## Discussion

### Dissolved oxygen, pH and phosphorus release

Phosphorus release from sediments has been extensively studied in freshwater systems [41, 42], and changes in the ability of sediments to sorb phosphorus (in the form of phosphate) is the classical explanation of variable release rates [43]. Sorption of phosphate prevents release, and occurs primarily in the presence of insoluble oxides of iron, manganese and aluminium [41, 42], and thus changes in the solubility of these oxides are a primary driver of desorption. Wang et al. [44] showed that in lentic systems under low oxygen conditions (2 to 4 mg L^-1^), phosphorus was released rapidly (within < 1 day), while in aerobic (> 4 to 7 mg L^-1^) and at oxygen saturation (> 7 mg L^-1^) there was negligible phosphorus release. Similarly, when pH is above a critical threshold (9.0 to 9.2), inorganic phosphorus desorbs from metal oxides at mineral surfaces [27, 28]. Cyanobacterial mats are well known for their steep and fluctuating physico-chemical gradients and the inner depths of the mats can experience anoxia and high pH [29]. However, to our knowledge, within *Phormidium*-dominated mats, DO and pH conditions have not been characterised in cobble-bedded rivers, and very little is known about DRP concentrations within cyanobacterial mat matrices [45].

In this study, DO measurements undertaken with micro-optodes showed that during the hours of daylight, when photosynthesis was occurring, concentrations within the mats exceeded those in the river, and declined to levels less than that of bulk water at night when photosynthesis ceased and respiration continued. When water flow was maintained across the *Phormidium* mats the within mat environment did not go anoxic, but there were marked periods (ca. 10 hours) during the night where within mat DO concentrations were less than 4 mg L^-1^. These sub-oxic conditions are conducive to the solubilisation of DRP from particulate material trapped within the *Phormidium* mat. It is possible that DO concentrations may have been lower still deeper within the mat, however, the fragility of the glass optodes prevented them being inserted to the base of the *Phormidium* mat. Malfunction of the mesocosm pump (between 02:00h and 05:30h, 13 March 2014) provided an opportunity to investigate DO concentrations within the mats in the absence of flow. During this period of static water the diffusive boundary layer (DBL) would have increased significantly, causing the diffusion rate of DO from the bulk water phase to decline. A decrease in the DO diffusion rate would account for the observed within mat DO concentration falling to 0 mg L^-1^. This illustrates the general interplay between flow, DBL thickness, mat thickness and solute fluxes associated with biofilms [16].

Although we only tracked pH within the mat during daylight hours, pH was above 9.0 for approximately 8 hours, while early morning values suggested that it had declined to circum-neutral overnight. As with DO, and as expected, the elevated pH was thus associated with the period of photosynthesis indicated by the PAM-derived rETR data. The pH in bulk river water was 7.0–8.0 indicating that the majority of dissolved carbon dioxide (CO_2_) is in the form of bicarbonate (HCO_3_^−^) [26]. Cyanobacteria are extremely efficient at utilising HCO_3_^−^, using carbonic anhydrase to converts it into CO_2_ and alkaline hydroxide ions (OH^−^) [46]. Accumulation of hydroxide ions results in increased pH in the micro-environment around the cell. This daytime elevation of pH, in concert with an extended sub-oxic period at night, suggests that conditions within the mat may be conducive to phosphate desorption for significant periods of the day. Based on our data, on a late summer’s day, conditions conducive to phosphorus -desportion may exist within *Phormidium* mats for up to 22 h.

Only very low nutrient concentrations are required during the initial stage of periphyton formation [47]. Transport of nutrients into mats depends on diffusion through the DBL that separates the developing mat from the bulk river water [48]. As mats continue to develop and become thicker the phosphorus demand increases. Cells within the mats may become nutrient-limited when nutrient consumption exceeds the capacity of diffusion across the DBL to supply community needs [49]. Our microelectrode data suggest that this boundary layer is of the order of 0.1 mm to 0.2 mm in the *Phormidium* mats investigated in this study. In addition to imposing nutrient limitations, boundary layer thickness also limits the diffusion rates of oxygen and hydroxide anions. They may thus facilitate the formation of DO or pH conditions within mats that are conducive to the release of phosphorus from trapped sediment.

The elevated levels of both DRP and iron in the within mat water are consistent with enhanced phosphorus-desorption from sediment trapped within and/or beneath the mats, as a result of low DO concentrations and/or high pH values. The DRP concentrations within the mat (ca. 380 μg L^-1^) were approximately 320-fold greater than the bulk river water. Not all of this observed difference in phosphorus will be due to release of sediment bound phosphate. We suggest extracellular enzymes with the ability to degrade organic matter, such as alkaline phosphatase (APA; [50]), also likely play an important role in within mat water phosphorus dynamics and this is an area that warrants further investigation. Evidence to support this comes from the identification of bacterial genera known to have APA activity in all successional stage of *Phormidium* mats [51]. In this study we have not consider organic phosphorus, however the ICP-MS analysis, which provides a measure of total phosphorus, suggests that this may also contribute to the elevated phosphorus load in the within mat water. Regardless of its origin, this increase in phosphorus in the within mat water creates an environment where this essential nutrient is not growth-limiting. In addition to increased release of P, iron is also known to stimulate cyanobacterial growth [52], and therefore solubilisation of iron-mineral phases (via formation of favourable DO and/or pH conditions) may further promote *Phormidium* growth.

### Photosynthesis

The rates of photosynthetic activity (as rETR) were highest in the early morning before declining after ca. 09:00h. Similar morning peaks followed by declines in photosynthetic rates (albeit usually later in the day ca. 12:00h) have been observed in biofilms and phytoplankton previously [53, 54]. This has been attributed to high dissolved inorganic carbon in the interstitial water as a result of respiration during the night. Photosynthetic CO_2_ fixation then depletes the dissolved inorganic carbon explaining the lower ETR measured—consistent with the elevation of DO and the decline in pH.

In this, study photosynthetic efficiency was less than 0.3 for most of the day, but returned to values of 0.7 overnight. Values of greater than 0.7 are generally expected of healthy cells [55]. The most likely explanation for this pattern is reversible photoinhibition, induced by a combination of high light and a limited supply of CO_2_ for use in carbon fixation [56]. In addition, like all optical instruments, the PAM devices used in this study only measure the cells on the mat surface. If this downregulation of activity is primarily caused by high light, cells in lower layers are protected due to shading by higher layers, and here photosynthesis may be occurring at higher rates for longer than on the surface. Values of *Y*_II_ fell below 0.1 at times, a situation where correlations between rETR and actual photosynthesis are fragile [57], and the best use of PAM data here is to indicate the period of active photosynthesis and we make no attempt to extrapolate to absolute rates.

### Sedimentation rates and biologically available phosphorus

If sediment provides a source of phosphorus for *Phormidium*, a relationship between sedimentation rates and the prevalence of *Phormidium* proliferations might be expected. This trend was observed by Wood and Bridge [24] who showed a correlation between increased quantity of deposited fine sediment and sites with *Phormidium* proliferations in the Maitai River (Nelson, New Zealand). In their study the site with the most extensive mats experienced 3.5-times higher sedimentation rates than the control site, and fine sediment (< 63 μm) accounted for ca. 70% of the total deposited sediment at sites with *Phormidium* proliferations.

To investigate whether sedimentation rates were related to the prevalence of *Phormidium* proliferations in the Mangatainoka River, sediment traps were deployed at three sites. A caveat when interpreting these data is that they only represent a limited time period and this might not be representative of the annual sedimentation characteristics of the sites. However, they do provide data over a period of prolific *Phormidium* growth at the lower two sites (B and C). The catchment of site A is largely native forest and no *Phormidium* proliferations have been observed (unpub. data, Horizons Regional Council). In contrast, the catchments of the two lower sites are dominated by agricultural land, and both experience regular proliferations, although these tend to be more common and extensive at site C (unpub. data, Horizons Regional Council). Our microscopic analysis indicated that larger particles were less likely to be incorporated into mats. A comparison of only the fine (< 63 μm) size fractions showed a significantly higher sedimentation rate at site C (the site with the most extensive proliferations), however there was no difference between sites A and B, suggesting that the difference in the quantity of deposited fine sediment may not be the sole reason for variations in *Phormidium* abundance among sites. Multiple physio-chemical factors are likely interacting and influencing *Phormidium* proliferations along the Mangatainoka River and there will be a hierarchy in importance among these. For example, during summer, dissolved inorganic nitrogen (DIN) at site A is below 0.2 mg L^-1^(www.lawa.org.nz), which is the generally accepted concentration required for bloom formation [14]. This may be the key factor that prevents *Phormidium* growth at this site.

In this study we considered three operationally-defined fractions of sediment-bound phosphorus that may be available to *Phormidium*: (1) Loosely adsorbed, which corresponds mostly to soluble inorganic phosphorus, and is mainly orthophosphate. This would be available to *Phormidium* under relatively neutral conditions. (2) Reductant soluble, which is assumed to be bound to iron-hydroxide and manganese oxide. When the within mat environment is in reductive state, due to low DO concentrations, this fraction of bound phosphorus is released. (3) Metallic oxide-bound phosphorus, which consists of phosphorus bound to iron and aluminium oxides. This can be released by a change of pH to higher values. In this study, metallic oxide-bound phosphorus was the most abundant form in the fine size classes. The observed increase in iron concentrations in the within mat water provides evidence to indicate this mechanism of P-release from sediment within *Phormidium* mats is occurring.

In addition to variations in sedimentation rates, the higher concentrations of the three phosphorus fractions at site C may partially explain differences in *Phormidium* abundance between the two lower sites. These sites are separated by less than 10 km and have similar surrounding land use. However, the < 63 μm, 63 μm and 125 μm fractions have almost double the amount of bound phosphorus. Reasons for these differences require further investigation but may include different land management practise e.g. fertiliser applications rates or cultivation.

## Conclusions

In this study we demonstrated that photosynthetic activity by natural mats of *Phormidium* can result in elevated pH (> 9.0), and that night-time respiration can cause DO depletion (< 4 mg L^-1^) within the mats. Within mat water typically contained 320-fold higher DRP concentrations than bulk river water. This, in concert with elevated concentrations of other elements (namely iron), suggest the solubilisation of more refractory pools of sediment-bound phosphorus. Sedimentation rates of fine sediment < 63 μm, were highest at the site with the most extensive proliferations, but did not differ significantly among the site with moderate proliferations and a site and where they never occur. Phosphorus fractionation demonstrated that there were markedly higher concentrations of potentially available phosphorus fractions at the two sites with *Phormidium* proliferations. Collectively these data provide compelling evidence that fine sediment provides a source of phosphorus that may contribute to *Phormidium* growth and proliferation.

## Supporting Information

S1 Table Average element (n = 9 for river and within *Phormidium* mat water), and dissolved reactive phosphorus (DRP; n = 16 (river water) and n = 15 (interstitial water)) concentrations in river water samples and within *Phormidium* mat water. Enrichment ratio = average interstitial concentration divided by average water concentration. All values are given in μg L^-1^. * Using a t-test the concentrations among the two water types were significantly different (p<0.01) from each other except for copper and cobalt.
(DOCX)

S2 Table Temporal variability (n = 1) in elements and dissolved reactive phosphorus (DRP) in river water from the Mangatainoka River (11 March to 12 March 2014. All values are given in μg L^-1^.
(DOCX)

S3 Table Temporal variability (n = 1) in elements and dissolved reactive phosphorus (DRP) in within *Phormidium* mat water (11 March to 12 March 2014). All values are given in μg L^-1^.
(DOCX)

## References

[1] Mohamed ZA, El-Sharouny HM, Ali WSM. Microcystin production in benthic mats of cyanobacteria in the Nile River and irrigation canals, Egypt. Toxicon 2006; 47: 584–590. 16564062 10.1016/j.toxicon.2006.01.029

[2] Heath MW, Wood SAG, Ryan K. Spatial and temporal variability in Phormidium and associated anatoxin production in two New Zealand rivers. Aquat. Microb. Ecol. 2011;64: 69–79.

[3] Quiblier C, Wood S, Echenique-Subiabre I, Heath M, Villeneuve A, Humbert J-F. A review of current knowledge on toxic benthic freshwater cyanobacteria—Ecology, toxin production and risk management. Wat. Res. 2013; 47: 5464–5479. 10.1016/j.watres.2013.06.042 23891539

[4] Krienitz L, Ballot A, Kotut K, Wiegand C, Pütz S, Metcalf J, et al Contribution of hot spring cyanobacteria to the mysterious deaths of Lesser Flamingos at Lake Bogoria, Kenya. FEMS Microbiol. Ecol. 2003;43: 141–148. 10.1111/j.1574-6941.2003.tb01053.x 19719674

[5] Gugger M, Lenoir S, Berger C, Ledreux A, Druart J-C, Humbert J-F, et al First report in a river in France of the benthic cyanobacterium *Phormidium favosum* producing anatoxin-a associated with dog neurotoxicosis. Toxicon. 2005;45: 919–928. 15904687 10.1016/j.toxicon.2005.02.031

[6] Wood SA, Heath MW, McGregor G, Holland PT, Munday R, Ryan KG. Identification of a benthic microcystin producing *Planktothrix* sp. and an associated dog poisoning in New Zealand. Toxicon. 2010;55: 897–903. 10.1016/j.toxicon.2009.12.019 20043936

[7] Faassen EJ, Harkema L, Begeman L, Lurling M. First report of (homo)anatoxin-a and dog neurotoxicosis after ingestion of benthic cyanobacteria in The Netherlands. Toxicon. 2012;60: 378–384. 10.1016/j.toxicon.2012.04.335 22534073

[8] Aboal M, Puig MA, Mateo P, Perona E. Implications of cyanophyte toxicity on biological monitoring of calcareous streams in north-east Spain. J Appl. Phycol. 2002;14: 49–56.

[9] Aboal M, Puig MA, Ríos H, López-Jiménez E. Relationship between macroinvertebrate diversity and toxicity of Cyanophyceae (Cyanobacteria) in some streams from eastern Spain. Verhandlungen des Internationalen Verein Limnologie. 2000;27: 555–559.

[10] Wood SA, Shearer K, Clapcott J. Advice on a monitoring programme to assess the ecological effects of Phormidium on macroinvertebrate communities. Cawthron Report No. 2624. 2014. 31 p.

[11] Wood SA, Selwood AI, Rueckert A, Holland PT, Milne JR, Smith KF, et al First report of homoanatoxin-a and associated dog neurotoxicosis in New Zealand. Toxicon. 2007;50: 292–301. 17517427 10.1016/j.toxicon.2007.03.025

[12] Wood SA, Heath M, Kuhajek J, Ryan K. Fine scale spatial variability of anatoxin-a and homoanatoxin-a production in benthic cyanobacteria; implication for monitoring and management. J. Appl. Microb. Ecol. 2010;109: 2011–2018. 10.1111/j.1365-2672.2010.04831.x 20722874

[13] Heath MW, Wood SA, Brasell K, Young R, Ryan KG. Development of habitat suitability criteria and in-stream habitat assessment for the benthic cyanobacteria *Phormidium* . Rivers Research Applications. 2015;31: 98–108.

[14] Wood SA, Wagenhoff A, Young R. The effect of flow and nutrients on Phormidium abundance and toxin production in rivers in the Manawatu-Whanganui region. Cawthron Report No. 2575. 2014. 42 p.

[15] Schindler DW, Hecky RE, Findlay DL, Stainton MP, Parker BR, Paterson MJ, et al Eutrophication of lakes cannot be controlled by reducing nitrogen input: results of a 37-year whole-ecosystem experiment. PNAS 2008;105: 11254–11258. 10.1073/pnas.0805108105 18667696 PMC2491484

[16] Stewart PS. Diffusion in biofilms. J. Bacteriol. 2003;185: 1485–1491. 12591863 10.1128/JB.185.5.1485-1491.2003 PMC148055

[17] Dodds W. The role of periphyton in phosphorus retention in shallow freshwater aquatic systems. J. Phycol. 2003;39: 840–849.

[18] Des Marais DJ. Marine hypersaline Microcoleus-dominated cyanobacterial mats in the saltern at Guerrero Negro, Baja California Sur, Mexico In: Seckbach J. editor. Microbial Mats. Springer Netherlands; 2010 pp 401–420.

[19] Revsbech NP. In situ measurement of oxygen profiles of sediments by use of oxygen microelectrodes In: Gnaiger E, Forstner H editor. Polarographic oxygen sensors. aquatic and physiological applications. Springer, Berlin, Heidelberg, New York: 1983; 265–273.

[20] Frantz CM, Petryshyn VA, Corsetti FA. Grain trapping by filamentous cyanobacterial and algal mats: implications for stromatolite microfabrics through time. Geobiology. 2015;13: 409–423. 10.1111/gbi.12145 26099298

[21] Pajdak-Stós A, Fiałkowska E, Fyda J. Phormidium autumnale (Cyanobacteria) defense against three ciliate grazer species. Aq. Microb. Ecol. 2001; 23:237–244.

[22] Hoiczyk E. Structural and biochemical analysis of the sheath of *Phormidium uncinatum* . J. Bacteriol. 1998;180: 3923–3932. 9683490 10.1128/jb.180.15.3923-3932.1998 PMC107377

[23] Kozerski HP. Determination of areal sedimentation rates in rivers by using plate sediment trap measurements and flow velocity—settling flux relationship. Wat. Res. 2002;36: 2983–2990. 10.1016/s0043-1354(01)00533-4 12171395

[24] Wood SA, Bridge B. Preliminary analysis of Phormidium abundance in the Maitai River and recommendation for on-going monitoring. Prepared for Nelson City Council. Cawthron Report No. 2537. 2014. 16 p.

[25] Pettersson K, Bostrȍm B, Jacobsen O. Phosphorus in sediments—speciation and analysis. Hydrobiol. 1988;170: 91–101.

[26] Zhou Q, Gibson CE, Zhu Y. Evaluation of phosphorus bioavailability in sediments of three contrasting lakes in China and the UK. Chemosphere 2001; 42: 221–225. 11237302 10.1016/s0045-6535(00)00129-6

[27] Andersen JM. Influence of pH on release of phosphorus from lake sediments, Archiv für Hydrobiol. 1975;76: 411–419.

[28] Eckert W, Nishri A, Parparova R. Factors regulating the flux of phosphate at the sediment-water interface of a subtropical calcareous lake: A simulation study with intact sediment cores. Wat. Air Soil Pol. 1997;99: 401–409.

[29] Brady A, Slater GF, Omelon CR, Southam G, Druschel G, Andersen A, et al Photosynthetic isotope biosignatures in laminated micro-stromatolitic and non-laminated nodules associated with modern, freshwater microbialites in Pavilion Lake, B.C. Chem. Geol. 2010: 274: 56–67.

[30] Stumm W, Morgan JJ. Aquatic Chemistry: Chemical Equilibria and Rates in Natural Waters, 3rd Edition Wiley-Interscience; 1995.

[31] Denis L, Gevaert F, Spilmont N. Microphytobenthic production estimated by in situ oxygen microprofiling: short-term dynamics and carbon budget implications. J. Soils and Sed. 2012: 1517–1529.

[32] APHA, AWWA and WEF. Standard Methods for the Examination of Water and Wastewater, 21st Edition American Public Health Association (APHA), American Water Works Association (AWWA), and Water Environment Federation (WEF); 2005.

[33] Hickey CW. Benthic chamber for use in rivers: testing against oxygen mass balances. J. Environ. Engineer. 1988;114: 828–845.

[34] Hofstraat JW, Peeters JCH, Snel JFH, Geel C. Simple determination of photosynthetic efficiency and photoinhibition of *Dunaliella tertiolecta* by saturating pulse fluorescence measurements. Mar. Ecol. Prog. Ser. 1994;103: 187–196.

[35] Hall K. The status of the river Dawros as a habitat for the freshwater pearl mussel Margaritifera margaritifera. MSc thesis. Trinity College, Dublin. 2008.

[36] R Core Team. R: A language and environment for statistical computing. R Foundation for Statistical Computing, Vienna, Austria 2014;URL http://www.R-project.org/.

[37] Anderson MJ A new method for non-parametric multivariate analysis of variance. Austral Ecology 2001;26:32–46.

[38] Lukkari K, Hartikainen H, Leivuori M. Fractionation of sediment phosphorus revisited. I: Fractionation steps and their biogeochemical basis. Limnol. Oceanogr. Methods 2007;5: 433–444.

[39] Psenner R, Pucsko R. Phosphorus fractionation: advantages and limits of the method for the study of sediment P origins and interactions. Arch. Hydrobiol. Beih 1988;30: 43–59.

[40] Xu D, Ding S, Li B, Bai X, Fan C, Zhang C. Speciation of organic phosphorus in a sediment profile of Lake Taihu I: Chemical forms and their transformation J. Enviro. Sci. 2013:25:637–644. 10.1016/s1001-0742(12)60136-3 23923771

[41] House WA. Geochemical cycling of phosphorus in rivers. Appl. Geochem. 2003;18: 739–748.

[42] Withers PJA, Jarvie HP. Delivery and cycling of phosphorus in rivers: A review. Sci. Tot. Environ. 2008;400: 379–395. 10.1016/j.scitotenv.2008.08.002 18804845

[43] Hupfer M, Lewandowski J. Oxygen controls the phosphorus release from lake sediments—a long-lasting paradigm. Internat. Rev. Hydrobiol. 2008;93: 415–432.

[44] Wang S, Jin X, Bu Q, Jiao L, Wu F Effects of dissolved oxygen supply level on phosphorus release from lake sediments. Colloids Surfaces A. 2008;316: 245–252.

[45] Stal LJ. Cyanobacterial mats and stromatolites In Whitton BA editor. The ecology of cyanobacteria. Springer; 2012: 65–125.

[46] Badger MR, Price GD. CO2 concentrating mechanisms in cyanobacteria: molecular components, their diversity and evolution. J. Exp. Bot. 2003;4: 609–622. 10.1093/jxb/erg076 12554704

[47] Biggs BJF. Use of relative specific growth rates of periphytic diatoms to assess enrichment of a stream. NZJ Mar. Freshwat. Res. 1990;24: 9–18.

[48] Jørgensen BB, Des Marais DJ. The diffusive boundary layer of sediments: oxygen micro-gradients over a microbial mat. Limnol. Oceanogr. 1990;35: 1343–1355. 11538698 10.4319/lo.1990.35.6.1343

[49] Bothwell ML. Phosphorus-limited growth dynamics of lotic periphytic diatom communities: Areal biomass and cellular growth rate response. Can. J. Fish. Aq. Sci. 1989;46: 1293–1301.

[50] Romani A, Sabater S. Influence of algal biomass on extracellular enzyme activity in river biofilms. Microb Ecol 2000;40: 16–24 10.1007/s002480000041 10977873

[51] Brasell K, Heath M, Ryan K, Wood S. Successional change in microbial communities of benthic Phormidium-dominated biofilms. Microb. Ecol. 2015;69: 254–266 10.1007/s00248-014-0538-7 25467742

[52] Li H, Murphy T, Guo J, Parr T, Nalewajko C. Iron stimulated growth and microcystin production of Microcystis novacekii UAM 250. Limnologica 2009; 39: 255–259.

[53] Pearl HW, Bebout BM, Prufert LE. Naturally occurring patterns of oxygenic photosynthesis and N2 fixation in marine microbial mats: Physiological and ecological ramification In: Cohen Y, Rosenberg E(editors). Microbial Mats—Physiological ecology of benthic microbial communities, ASM, Washington; 1989 pp 326–341.

[54] Storch TA, Saunders GW, Ostrofsky ML. Diel nitrogen fixation by cyanobacterial surface blooms in sanctuary lake, Pennsylvania. Appl. Environ. Microbiol. 1990: 56: 466–471. 16348120 10.1128/aem.56.2.466-471.1990 PMC183362

[55] Beuzenberg V, Smith KF, Packer MA Isolation and characterisation of halo-tolerant Dunaliella strains from Lake Grassmere/Kapara Te Hau, New Zealand. NZ J. Bot. 2014; 52: 136–152.

[56] Tyystjärvi E. Photoinhibition of photosystem II. Int. Rev. Cell. Mol. Biol. 2013;300: 243–303. 10.1016/B978-0-12-405210-9.00007-2 23273864

[57] Beer S, Axelsson L. Limitations in the use of PAM fluorometry for measuring photosynthetic rates of macro-algae at high irradiances. Eur. J. Phycol. 2004;39: 1–7.

