# Supplementary material for: Entrapped Sediments as a Source of Phosphorus in Epilithic Cyanobacterial Proliferations in Low Nutrient Rivers
Source: PLoS One. 2015 Oct 19;10(10):e0141063. doi: 10.1371/journal.pone.0141063 (PMC4610676; doi:10.1371/journal.pone.0141063)
Supplement: S1 Table — Enrichment ratio = average interstitial concentration divided by average water concentration. All values are given in μg L-1. * Using a t-test the concentrations among the two water types were significantly different (p<0.01) from each other except for copper and cobalt. (DOCX) [file pone.0141063.s001.docx]

**S1 Table.** Average element (n = 9 for river and within *Phormidium* mat water), and dissolved reactive phosphorus (DRP; n = 16 (river water) and n = 15 (interstitial water)) concentrations in river water samples and within *Phormidium* mat water. Enrichment ratio = average interstitial concentration divided by average water concentration. All values are given in µg L^-1^. * Using a t-test the concentrations among the two water types were significantly different (p<0.01) from each other except for copper and cobalt.

|  | **Interstitial water** | | **River water** | | **Enrichment** |
| --- | --- | --- | --- | --- | --- |
| **Element / nutrient** | **Average** | **Stdev** | **Average** | **Stdev** | **ratio** |
| DRP | 381 | 16.0 | 1.2 | 1 | 320 |
| [Phosphorus](http://en.wikipedia.org/wiki/Phosphorus) | 441 | 14.3 | 3.8 | 1.0 | 115 |
| [Cadmium](http://en.wikipedia.org/wiki/Cadmium) | 0.08 | 0.03 | 0.002 | 0.002 | 37 |
| [Arsenic](http://en.wikipedia.org/wiki/Arsenic) | 0.48 | 0.10 | 0.01 | 0.02 | 33 |
| Iron | 57.0 | 38.8 | 2.7 | 2.2 | 21 |
| Potassium | 15,207 | 3,705 | 1,159 | 49 | 13 |
| Nickel | 2.1 | 0.5 | 0.2 | 0.0 | 11 |
| Boron | 442 | 162 | 55 | 59 | 8 |
| Aluminium | 33.3 | 15.8 | 7.2 | 1.4 | 5 |
| Zinc | 75 | 33 | 17 | 12 | 4 |
| [Boron](http://en.wikipedia.org/wiki/Boron) | 87.0 | 22.2 | 20.4 | 3.5 | 4 |
| [Chromium](http://en.wikipedia.org/wiki/Chromium) | 1.4 | 0.3 | 0.4 | 0.1 | 4 |
| Manganese | 5.0 | 2.2 | 1.7 | 0.5 | 3 |
| Lead | 0.3 | 0.2 | 0.2 | 0.1 | 2 |
| [Vanadium](http://en.wikipedia.org/wiki/Vanadium) | 0.5 | 0.2 | 0.3 | 0.1 | 2 |
| Magnesium | 2,808 | 537 | 1,723 | 59 | 2 |
| Sodium | 11,180 | 1,025 | 7,042 | 161 | 2 |
| Calcium | 12,751 | 2,033 | 8,148 | 128 | 2 |
| Strontium | 120 | 17.0 | 77 | 4.4 | 2 |
| Copper* | 9.3 | 3.2 | 7.0 | 2.1 | 1 |
| Cobalt* | 0.7 | 0.4 | 0.5 | 0.5 | 1 |
| [Silicon](http://en.wikipedia.org/wiki/Silicon) | 3,986 | 299 | 3,576 | 112 | 1 |
| [Selenium](http://en.wikipedia.org/wiki/Selenium) | 0.1 | 0.1 | 0.0 |  |  |
