# Supplementary material for: Entrapped Sediments as a Source of Phosphorus in Epilithic Cyanobacterial Proliferations in Low Nutrient Rivers
Source: PLoS One. 2015 Oct 19;10(10):e0141063. doi: 10.1371/journal.pone.0141063 (PMC4610676; doi:10.1371/journal.pone.0141063)
Supplement: S2 Table — All values are given in μg L-1. (DOCX) [file pone.0141063.s002.docx]

S2 Table. Temporal variability (n = 1) in elements and dissolved reactive phosphorus (DRP) in river water from the Mangatainoka River (11 March to 12 March 2014. All values are given in µg L^-1^.

|  | **Time** | **B** | **Na** | **Mg** | **Al** | **Si** | **P** | **K** | **Ca** | **V** | **Cr** | **Fe** | **Mn** | **Co** | **Ni** | **Cu** | **Zn** | **As** | **Sr** | **Cd** | **Ba** | **Hg** | **Pb** | **DRP** |
| --- | --- | --- | --- | --- | --- | --- | --- | --- | --- | --- | --- | --- | --- | --- | --- | --- | --- | --- | --- | --- | --- | --- | --- | --- |
| **11/03/2014** | **11:00** | 52 | 10206 | 2730 | 40 | 3918 | 572 | 17384 | 12192 | 0.40 | 1.45 | 56 | 3.38 | 0.27 | 1.86 | 6.46 | 64 | 0.44 | 109 | 0.11 | 669 | 0.18 | 0.23 | 1 |
| **11/03/2014** | **13:00** | 67 | 11336 | 2253 | 32 | 3890 | 456 | 12397 | 10457 | 0.84 | 1.75 | 74 | 3.16 | 0.32 | 2.25 | 6.38 | 39 | 0.40 | 105 | 0.08 | 649 | 0.04 | 0.49 | 1 |
| **11/03/2014** | **15:30** | 119 | 12689 | 2928 | 65 | 4510 | 602 | 14142 | 12939 | 0.88 | 1.94 | 132 | 5.11 | 0.34 | 2.81 | 6.89 | 68 | 0.47 | 112 | 0.12 | 455 | 0.05 | 0.68 | 2 |
| **11/03/2014** | **17:30** | 103 | 11790 | 2769 | 47 | 4256 | 596 | 13184 | 12768 | 0.58 | 1.45 | 103 | 4.22 | 0.31 | 2.32 | 8.22 | 53 | 0.64 | 118 | 0.12 | 541 | 0.04 | 0.33 | 1 |
| **11/03/2014** | **19:00** | 76 | 10936 | 3762 | 20 | 4158 | 351 | 14917 | 16744 | 0.34 | 1.39 | 35 | 3.24 | 0.71 | 1.70 | 10.32 | 41 | 0.48 | 154 | 0.07 | 295 | 0.03 | 0.24 | 2 |
| **11/03/2014** | **00:00** | 93 | 9716 | 2351 | 15 | 3676 | 198 | 12945 | 11168 | 0.38 | 1.02 | 13 | 8.94 | 0.83 | 1.12 | 10.08 | 110 | 0.39 | 113 | 0.03 | 185 | 0.02 | 0.22 | 2 |
| **12/03/2014** | **07:30** | 113 | 10177 | 2231 | 17 | 3648 | 375 | 10309 | 10781 | 0.32 | 1.15 | 29 | 8.15 | 1.32 | 2.79 | 16.78 | 138 | 0.54 | 105 | 0.05 | 409 | 0.02 | 0.32 | 2 |
| **12/03/2014** | **09:30** | 73 | 11346 | 2716 | 30 | 3829 | 512 | 20348 | 12697 | 0.43 | 1.30 | 40 | 4.82 | 1.04 | 1.89 | 10.28 | 93 | 0.61 | 127 | 0.08 | 462 | 0.03 | 0.25 | 2 |
| **12/03/2014** | **11:30** | 89 | 12426 | 3528 | 35 | 4600 | 303 | 21236 | 15017 | 0.59 | 1.47 | 31 | 3.65 | 0.89 | 2.14 | 8.11 | 68 | 0.35 | 141 | 0.08 | 317 | 0.03 | 0.28 | 2 |
