# Supplementary material for: Entrapped Sediments as a Source of Phosphorus in Epilithic Cyanobacterial Proliferations in Low Nutrient Rivers
Source: PLoS One. 2015 Oct 19;10(10):e0141063. doi: 10.1371/journal.pone.0141063 (PMC4610676; doi:10.1371/journal.pone.0141063)
Supplement: S3 Table — All values are given in μg L-1. (DOCX) [file pone.0141063.s003.docx]

S3 Table. Temporal variability (n=1) in elements and dissolved reactive phosphorus (DRP) in within *Phormidium* mat water (11 March to 12 March 2014). All values are given in µg L^-1^.

|  | **Time** | **B** | **Na** | **Mg** | **Al** | **Si** | **P** | **K** | **Ca** | **V** | **Cr** | **Fe** | **Mn** | **Co** | **Ni** | **Cu** | **Zn** | **As** | **Sr** | **Cd** | **Ba** | **Hg** | **Pb** | **DRP** |
| --- | --- | --- | --- | --- | --- | --- | --- | --- | --- | --- | --- | --- | --- | --- | --- | --- | --- | --- | --- | --- | --- | --- | --- | --- |
| **11/03/2014** | **11:00** | 20 | 6734 | 1701 | 9 | 3454 | 4 | 1175 | 8086 | 0.38 | 0.29 |  | 1.70 | 0.13 | 0.18 | 5.75 | 15 |  | 76 |  | 33 | 0.02 | 0.17 | 522 |
| **11/03/2014** | **13:00** | 18 | 7139 | 1797 | 7 | 3695 | 3 | 1188 | 8262 | 0.33 | 0.41 |  | 1.55 | 0.10 | 0.16 | 4.10 | 8 |  | 71 |  | 31 | 0.02 | 0.29 | 450 |
| **11/03/2014** | **15:30** | 17 | 7177 | 1800 | 8 | 3687 | 4 | 1175 | 8145 | 0.35 | 0.39 |  | 1.35 | 0.15 | 0.18 | 5.63 | 11 |  | 71 | 0.01 | 33 | 0.00 | 0.17 | 536 |
| **11/03/2014** | **17:30** | 27 | 7237 | 1663 | 8 | 3543 | 4 | 1063 | 8103 | 0.32 | 0.62 |  | 1.09 | 0.14 | 0.21 | 6.12 | 48 |  | 77 |  | 212 | 0.02 | 0.13 | 564 |
| **11/03/2014** | **19:00** | 19 | 7157 | 1715 | 9 | 3539 | 4 | 1181 | 7967 | 0.35 | 0.30 | 0.6 | 1.11 | 0.45 | 0.25 | 6.44 | 12 |  | 77 |  | 31 | 0.01 | 0.15 | 323 |
| **11/03/2014** | **00:00** | 21 | 6953 | 1678 | 6 | 3525 | 3 | 1160 | 8034 | 0.30 | 0.35 |  | 2.06 | 0.61 | 0.18 | 7.06 | 14 | 0.04 | 81 |  | 35 | 0.01 | 0.12 | 203 |
| **12/03/2014** | **07:30** | 16 | 7097 | 1794 | 6 | 3762 | 6 | 1232 | 8319 | 0.18 | 0.44 | 4.9 | 2.36 | 1.17 | 0.19 | 11.21 | 18 |  | 75 |  | 34 | 0.01 | 0.19 | 312 |
| **12/03/2014** | **09:30** | 21 | 6907 | 1697 | 6 | 3524 | 3 | 1153 | 8330 | 0.19 | 0.35 |  | 2.18 | 0.91 | 0.18 | 7.66 | 13 |  | 81 | 0.01 | 34 | 0.01 | 0.11 | 457 |
| **12/03/2014** | **11:30** | 24 | 6975 | 1658 | 6 | 3451 | 4 | 1105 | 8085 | 0.17 | 0.32 | 2.5 | 1.82 | 1.24 | 0.16 | 9.33 | 14 | 0.02 | 84 |  | 49 | 0.01 | 0.17 | 272 |
